
JOURNAL INFORMATION
==============================
NLM Title Abbreviation: ScientificWorldJournal
Journal ID: ScientificWorldJournal
NLM Journal ID: 101131163
Journal ID: TSWJ

The Scientific World Journal

ISSN: 2356-6140
EISSN: 1537-744X
Publisher: Wiley

ARTICLE INFORMATION
==============================
PMCID: PMC6084152
PMID: 30147591
DOI: 10.1100/tsw.2001.127
Article ID: 690735
Article version: 1
Subjects: Short Report

Inhibition of Human Telomerase Reverse Transcritpase (hTERT) and Telomerase Activity by Curcumin in Human Mammary Epithelial and Breast Carcinoma Cell Lines

Ramachandran Chappail 1 2 *
Fonseca Hugo B. 2
Jhabvala Perseus 1
Melnick Steven J. 1
Escalon Enrique 3
Department of Pathology, Miami Children’s Hospital, 3100 SW 62 Avenue, Miami, FL 33155, USA
Research Institute, Miami Children’s Hospital, 3100 SW 62 Avenue, Miami, FL 33155, USA
Department of Hematology and Oncology, Miami Children’s Hospital, 3100 SW 62 Avenue, Miami, FL 33155, USA
*Corresponding author.
Publication date: 2001
Electronic publication date: 2001 Oct 17
Volume: 1
First page: 109
Last page: 109
Copyright: Copyright © 2001 Chappail Ramachandran et al.
Copyright year: 2001
License: This is an open access article distributed under the Creative Commons Attribution License, which permits unrestricted use, distribution, and reproduction in any medium, provided the original work is properly cited.
License URL: https://creativecommons.org/licenses/by/3.0/

==============================
Miami *Nature Biotechnology* Short Reports
*TheScientificWorld* (2001) 1(S3), 109SR
ISSN 1532-2246; DOI 10.1100/TSW.2001.127

### INHIBITION OF HUMAN TELOMERASE REVERSE TRANSCRITPASE (HHERT) AND TELOMERASE ACTIVITY BY CURCUMIN IN HUMAN MAMMARY EPITHELIAL AND BREAST CARCINOMA CELL LINES

Cheppail Ramachandran[1,2,*], Hugo B. Fonseca[2], Perseus Jhabvala[1], Steven J. Melnick[1], and Enrique Escalon[3]

[1]Department of Pathology, [2]Research Institute, [3]Hematology and Oncology, Miami Children's Hospital, 3100 SW 62 Avenue, Miami, FL 33155
* Corresponding author.

**INTRODUCTION.** Telomeres are the distal ends of chromosomes composed of tandem repeats of the sequence TTAGGG. Several lines of evidence have indicated that activation of telomerase enzyme and telomere stabilization are important necessary steps in tumorogenesis (1,2). Telomerase activity is usually repressed in somatic cells, but is reactivated in immortal cells and human cancers. Human telomerase has two components, an RNA (hTER) which serves as template for the simple sequence and the catalytic subunit that acts as reverse transcriptase (hTERT). In most cases hTERT has been found to be the limiting factor for telomerase activity (3). Inhibition of hTERT results in telomere loss and limits the growth of tumor cells. Furthermore, these cells undergo apoptosis when their telomeres reach a critically short length, which usually occurs after several generations. Telomerase has been frequently described as an ideal cancer target because it is activated in most cancer cells (2,4). Curcumin (diferuloyl methane) the major yellow-colored dietary pigment from the rhizomes of turmeric (*Curcuma longa* L.) has anti-inflammatory, anti-tumor, anti-proliferative and anti-oxidant properties. Curcumin is a potent inhibitor of mutagenesis and chemically induced carcinogenesis in animal tumor models (5,6). In a recent study, we reported that curcumin can induce a high percentage of apoptosis in breast carcinoma cells, but the same concentration of curcumin caused an insignificant level of apoptosis in human mammary epithelial cells. Curcumin down-regulates Ki67, PCNA and mutant p53 mRNAs in breast cancer cells, these properties may underlie chemopreventive action (6). In this investigation, we analyzed the mechanism underlying the inhibition of telomerase activity by curcumin and the genes associated with the telomere complex in breast carcinoma cell lines.

**METHODS.** Human mammary epithelial cell line (MCF-10A), drug sensitive breast carcinoma (MCF-7) and multidrug resistant breast carcinoma (MCF-7/TH) cell lines were grown in DMEM medium supplemented with fetal bovine serum, L-glutamine and antibiotics. Curcumin cytotoxicity was determined by a growth inhibition assay using trypan blue stain and $IC_{50}$ values were determined (7). Curcumin-induced apoptosis was estimated by terminal dideoxynucleotidyl transferase (TdT) assay using the *in situ* cell death detection (TUNEL) kit and the cellular FITC fluorescence was analyzed in a Coulter Elite flow cytometer (8). Telomerase activities in curcumin-treated and -untreated cells were analyzed using TRAPEZE kit from Intergen Co., NY. To determine the effect of curcumin on hTERT and hTER gene expression, RT-PCR assay was performed with total RNA (0.5 µg) using Perkin-Elmer rTth EZ RNA-PCR kit and gene specific sense and antisense primers. RT-PCR products were electrophoresed on 2% Nu-Sieve agarose gel and relative expression estimated by densitometry. Beta-actin mRNA was also amplified as control in the RT-PCR assay.

**RESULTS AND DISCUSSION.** Fig. 1 shows curcumin cytotoxicity curves of human mammary epithelial (MCF-10A) and breast carcinoma cell lines (MCF-7 and MCF-7/TH). Curcumin $IC_{50}$ values indicated that breast carcinoma cell lines were more sensitive to curcumin than human mammary epithelial cell line. In short, breast cancer cells have collateral sensitivity to curcumin. Cytotoxicity data correlated with the percentages of apoptosis induced by curcumin. The percentage of apoptotic cells induced by curcumin is significantly higher in breast carcinoma cell lines than mammary epithelial cell line. Telomerase activity in breast carcinoma cell lines was 5.7 fold (MCF-7, Absorbance = 2.71) and 6.9 fold (MCF-7/TH, Absorbance = 3.31) higher than that in human mammary epithelial cell line (MCF-10, Absorbance = 0.48). Curcumin treatment (100 µM) of breast carcinoma cells inhibited 67% and 94% telomerase activity in MCF-7 and MCF-7/TH cells, respectively (Fig.2 & 3). However, curcumin treatment did not induce any change in the telomerase activity of MCF-10A cells. Curcumin downregulates hTERT and not hTER expression in breast carcinoma cells compared to no change in the expression of hTERT and hTER in human mammary epithelial cell line (Fig. 4).

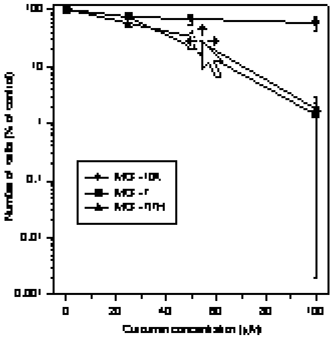

**Fig. 1.** Curcumin cytotoxicity of human mammary epithelial (MCF-10A) and breast carcinoma (MCF-7 and MCF-7/TH) cell lines.

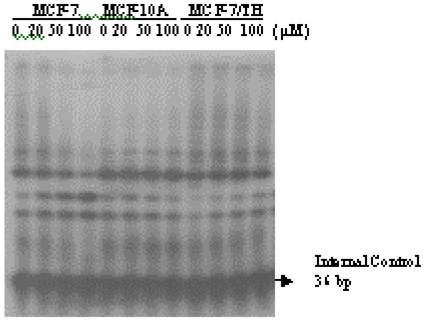

**Fig. 2.** Gel analysis of telomerase activity in tumor cells.

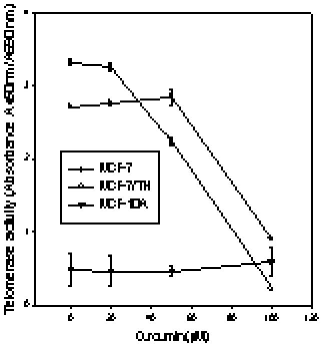

**Fig. 3.** Inhibition of telomerase activity by curcumin.

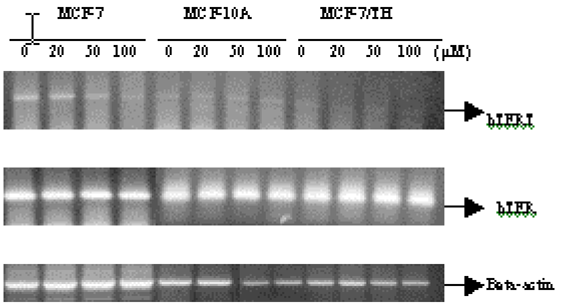

**Fig. 4.** hTERT and hTER expression in curcumin treated human mammary epithelial and breast carcinoma cells.

**CONCLUSION.** The chemopreventive action of curcumin against human malignancies may involve repression of telomerase activity in tumor cells, one of the major factors in carcinogenesis. Inhibition of telomerase activity by curcumin involves down regulation of hTERT mRNA expression in breast carcinoma cell lines.

**REFERENCES.**
1. Greider, C.W. (1999) Telomerase activation - one step on the road to cancer. Trends Genet. 15, 109-112
2. Buys, C.H.C.M. (2000) Telomeres, telomerase and cancer. N. Engl. J. Med. 342, 1282-1283
3. Greider, C.W. (1998) Telomerase activity, cell proliferation and cancer. Proc. Natl. Acad. Sci. U S A 95, 90-92
4. Kim, N.M. Clinical implications of telomerase in cancer. Eur. J. Cancer 33, 781-786
5. Huang, M.T., Lou, Y.R., Xie, J.G., Ma, W., Lu, Y.P., Yen, P., Zhu, B.T., Newmark, H., and Ho, C.T. (1998) Carcinogenesis 19, 1697-1700
6. Ramachandran, C. and You, W. (1999) Breast Cancer Res. Treat. 54, 269-278
7. Kawamori, T., Lubet, R., Steele, V.E., Kelloff, G.J., Kaskey, R.B., Rao, C.V., and Reddy, B.S. (1999) Cancer Res. 59, 597-560
8. Ramachandran, C., You, W., and Krishan, A. (1998) Anticancer Res. 17, 3369-3376
